# Excessive Smoke from a Neighborhood Restaurant Highlights Gaps in Air Pollution Enforcement: Citizen Science Observational Study

**DOI:** 10.3390/air3030020

**Published:** 2025-07-18

**Authors:** Nicholas C. Newman, Deborah Conradi, Alexander C. Mayer, Cole Simons, Ravi Newman, Erin N. Haynes

**Affiliations:** 1 Department of Pediatrics, College of Medicine, University of Cincinnati, Cincinnati, OH, 45229, USA; 2 Division of General and Community Pediatrics, Cincinnati Children’s Hospital Medical Center, Cincinnati, OH, 45229, USA; 3 Department of Environmental and Public Health Sciences, College of Medicine, University of Cincinnati, Cincinnati, OH, 45267, USA;; 4 Independent Researcher Cincinnati, OH, 45231, USA;; 5 College of Medicine, University of Cincinnati, Cincinnati, OH, 45267, USA;; 6 Department of Political Science, College of Arts and Sciences, Ohio State University, Columbus, OH, 43210, USA;

**Keywords:** air pollution, citizen science, environmental regulation

## Abstract

Regulatory air pollution monitoring is performed using a sparse monitoring network designed to provide background concentrations of pollutants but may miss small area variations due to local emission sources. Low-cost air pollution sensors operated by trained citizen scientists provide an opportunity to fill this gap. We describe the development and implementation of an air pollution monitoring and community engagement plan in response to resident concerns regarding excessive smoke production from a neighborhood restaurant. Particulate matter (PM_2.5_) was measured using a low-cost, portable sensor. When cooking was taking place, the highest PM_2.5_ readings were within 50 m of the source (mean PM_2.5_ 36.9 μg/m^3^) versus greater than 50 m away (mean PM_2.5_ 13.0 μg/m^3^). Sharing results with local government officials did not result in any action to address the source of the smoke emissions, due to lack of jurisdiction. A review of air pollution regulations across the United States indicated that only seven states regulate food cookers and six states specifically exempted cookers from air pollution regulations. Concerns about the smoke were communicated with the restaurant owner who eventually changed the cooking fuel. Following this change, less smoke was observed from the restaurant and PM_2.5_ measurements were reduced to background levels. Although current environmental health regulations may not protect residents living near sources of food cooker-based sources of PM_2.5_, community engagement shows promise in addressing these emissions.

## 1. Introduction

Fine particulate matter (PM_2.5_) is one of five criteria air pollutants regulated by The Clean Air Act and has well-known respiratory, cardiovascular, and neurologic health effects [1–4]. Air pollution monitoring for PM_2.5_ uses a monitoring network that is designed to provide background concentrations of PM_2.5_ [5]. Due to a variety of factors, these monitoring stations are often limited in number and are unable to characterize neighborhood-level gradients in exposure due to point sources [6]. There is evidence demonstrating that other non-industrial sources, such as restaurants, may be significant contributors to local or neighborhood levels of PM_2.5_ air pollution [7–11]. Based on measurement of emission plumes in Las Vegas, NV with supplemental data from Los Angeles, CA, and Boulder, CO, restaurant sources may contribute up to 20% of the anthropogenic volatile organic compounds in those areas [12]. Comparing the type of cooking (bakery versus bar-b-que) suggests higher variability in particle counts with bar-b-que cooking [13]. Investigators from Pittsburgh noted that restaurant emissions can create daily increases in particulate matter in restaurant districts after the emissions associated with vehicular transport for commuting have decreased [14]. Thus, those who live near these establishments may be exposed to higher average levels of air pollution and experience peaks of exposure that the general population does not.

Citizen science, defined as “the involvement of the public in scientific research,” typically involves projects in which members of the public partner with scientists to answer real-world questions [15]. With the development of lower-cost air pollution sensors, citizen science is one approach to address gaps in air pollution exposure assessment at a neighborhood level [16,17]. Pairing interested citizens with academic researchers is one way to empower citizens with the knowledge and equipment to conduct citizen science (e.g., air pollution sensors, training, data analysis capabilities) [17,18].

The National Institute for Environmental Health Sciences (NIEHS) funds a network of Environmental Health Sciences Core Centers, each with a Community Engagement Core (CEC) “to foster community–university partnerships.” [19]. The CEC funds are not meant for environmental health research. The University of Cincinnati’s CEC has a history of working with Citizen Scientists and engaging communities on monitoring and environmental health dissemination projects [20–23]. The CEC partners with communities on projects in three domains: (1) environmental health research translation, (2) participant engagement, or (3) socio-ecological systems (policy change, building community capacity) [20–24].

In the summer of 2014, a local resident became concerned about air quality near their home when a new restaurant moved into their neighborhood. There were clouds of smoke extending beyond the fenced outdoor cooking area. The restaurant is approximately 350 m from their home. Anecdotal reports of smoke and odors penetrating homes with windows closed, respiratory symptoms, and eye irritation that improved when not in the home resulted in air quality complaints to the municipality and the Southwest Ohio Air Quality Agency in 2014 and 2015. The Agency noted clouds of smoke drifting over the neighborhood from the fenced-in cooking area in the back of the restaurant. The Southwest Ohio Air Quality Agency investigated the smoke but could not act because existing regulations from Ohio EPA did not provide air permits to restaurant cookers. Lack of action prompted a community member to contact the University of Cincinnati NIEHS-funded CEC for assistance and objectives were articulated (Table 1). To assist the community member in reducing pollution from the source, we also reviewed state policies regulating emissions from cooking sources.

The objective of this project was to create and implement a community–academic partnership by working with a concerned community member to objectively measure PM_2.5_ levels in the vicinity of a local restaurant and to advocate for reduction in emissions. This data would be presented to the proprietor of the restaurant, local regulatory authorities, and the community at large to demonstrate the problem and to stimulate additional action. This paper is a case report of this project.

## 2. Materials and Methods

### 2.1. Study Location

The study location is a suburb of Cincinnati, Ohio approximately 15 miles north of downtown Cincinnati, Ohio, with a population of approximately 6850 people. The community has approximately 3000 households with a median household income of USD 45,546 [25]. It ranks twenty-sixth-highest out of ninety-one communities in Hamilton County for childhood admissions for asthma at the regional children’s hospital [26]. According to US EPA Environmental Justice Screen Tool, it is in the eighty-seventh percentile in the US for PM_2.5_, with an annual mean PM_2.5_ of 10.3 μg/m^3^ [27]. The restaurant is located in the downtown business district and prepares grilled meats cooked using a charcoal fired outdoor commercial grill and bar-b-que foods. To assess other potential sources of PM_2.5_, a manual review of a 200 m radius circle centered on the location of the restaurant, created using Google Maps and Federal Communications Commission Circleplot [28], was used to identify businesses close to the restaurant. Potential sources of PM_2.5_ emissions within that circle were a US Post Office, seven restaurants, three bus stops, one auto repair shop, and five offices for construction/maintenance companies (Table 2). The restaurant was within 40 m of a major street. Additionally, US EPA daily average PM2.5 data for the four closest monitors (7.25 km–12.63 km distance from the restaurant) from the US EPA website https://www.epa.gov/outdoor-air-quality-data/download-daily-data (accessed on 10 February 2025) were used to provide measured ambient PM_2.5_ from a “gold standard” monitor. If the US EPA monitor reported more than one reading for a given day, the average of these readings was taken as the reading for that day. The readings from the four closest monitors were used because not all monitors were working on all days that readings were collected using the portable sensor.

### 2.2. Air Quality Assessment Protocol

To quantify the ambient air pollution levels in the vicinity of the BBQ restaurant of concern, an air monitoring protocol was developed. Since PM_2.5_ is an expected pollutant from grilling and burning food [29], air pollution was measured using the AirBeam by HabitatMap (HabitatMap, Brooklyn, NY, 11217, USA), a portable, low-cost, manufacturer-calibrated, user-friendly real-time sensor. The AirBeam measures PM_2.5_ using a light scattering method that has demonstrated good correlation with reference methods [30]. The AirBeam was paired by Bluetooth^®^ with an Android (“Android” is a trademark of Google LLC, Mountain View, CA, 94043, USA) mobile device via the open source AirCasting app [31]. Each monitoring session output a datafile with PM_2.5_ measurements averaged over one second as well as a map (displayed on the Android device), overlaid with color-coded dots that correspond to the US EPA Air Quality Index (AQI) for PM_2.5_ at the location based on the global-positioning-system coordinates from the Android mobile device. The software calculated the average and peak PM_2.5_ measured during each session.

Based on the expected dispersion of PM_2.5_, the monitoring plan consisted of the community member carrying the AirBeam on a walking route from their home located approximately 350 m south of the restaurant towards the restaurant. The route also included approximately two blocks in each direction (north, south, east, west) along the sidewalks of local streets (public right of way) adjacent to the source restaurant in question. This route placed the restaurant in the center with additional monitoring taken approximately three-hundred meters in each direction, then returning home. Sessions occurred between 7AM and 7PM on days that the community member was available. Over the course of the project, there were sessions spread across seven days of the week. The community member walked with the sensor held at approximately chest-height with the inlet exposed, typically on days when there was not heavy rain. Average daily windspeed and wind direction were obtained from National Oceanic and Atmospheric Administration records for the monitoring days. Air pollution monitoring sessions were carried out by a single community member with a single sensor. The sessions took place when the community member was available to carry them out. Sensor data was collected from June 2016-June 2017. This allowed us to characterize the emission source and compare it with the surrounding neighborhood. For quality assurance, the academic partner provided training and was present for the first air monitoring session and available periodically during the process to help the community member with the device. The community partner conducted the remaining seventeen sessions. One additional session was conducted approximately one-thousand meters away to assess background levels of PM_2.5_.

In April 2024, members of the team returned to the neighborhood to measure PM_2.5_ emissions due to reports from the community member that the smoke had improved over the previous two years. Since the original AirBeam sensors were not functional after several years, these sessions used newer AirBeam3 sensors (HabitatMap Brooklyn, NY, 11217, USA). To collect additional background PM_2.5_, there were stationary sessions conducted as well. Members from the academic partner met with the community member to plan out the monitoring and instructed on the use of the sensor and to perform quality assurance with a companion monitor. They were available to the community member to provide technical support, feedback on results, and to analyze the data.

### 2.3. Statistical Analysis

Analysis and visualization were carried out by the academic partner. Measurements from the AirBeam sensors (PM_2.5_ and latitude, longitude coordinates) were transferred to Microsoft Excel. Missing data was not included in analysis. Distance between the restaurant location and sensor measurements was calculated using the “Great Circle Distance” calculator for Microsoft Excel [32], descriptive statistics (mean, standard deviation) were calculated, and the distance from the restaurant vs. PM_2.5_ levels were analyzed. One minute average PM_2.5_ was calculated to align values with the US EPA AQI Pilot 1 min guidance for low-cost personal air quality monitors [33]. The ArcGIS for Microsoft 365 Excel Add-In (Esri, Redlands, CA, 92373, USA) was used to create visualizations of PM_2.5_ measurements from air pollution monitoring sessions. Since the monitoring sessions were mobile (conducted whilst walking in the neighborhood), bearing relative to the restaurant and relative to the wind were calculated (using Microsoft Excel’s IMARGUMENT function) to determine when the sensor was downwind (sensor bearing 135°–225° relative to the restaurant and wind direction). To test the hypothesis that PM_2.5_ readings would be higher closer to the restaurant, Pearson correlation between PM_2.5_ and distance was calculated. This was further tested by adjusting for average daily windspeed, and relative position (downwind or upwind) in a multiple linear regression model. Wizard for Mac v2.0.19 (Evan Miller) was used for statistical analyses.

### 2.4. Dissemination Plan

The dissemination plan focused on the socio-ecological systems (policy change, building community capacity) and research domains of the project. We distributed a PM_2.5_ and health fact sheet at a local health fair (April 2017) and attendees were invited to play an educational air pollution dice game. The results from the initial set of air monitoring sessions were summarized for the health fair and were communicated to relevant governmental agencies and elected officials (Hamilton County Auditor, Hamilton County Commissioners Office, Hamilton County Public Health, Ohio EPA, and US EPA). During this project, the owner of the restaurant and the community member communicated and discussed the smoke emissions, including identifying potential interventions (e.g., changing the fuel used for grilling from charcoal to wood, addition of exhaust fans, etc.) that could reduce community exposure.

### 2.5. Policy Review

To review air pollution regulations state-by-state with regard to cooking emissions, a search of state air quality agency websites was performed. Specifically, a search of “food”, “cook”, “broiler”, “roaster”, “charboiler” or “cooker” was made within the permits or permitting requirements for each state’s air quality agency. If it was not found, then a search within the state law database would be performed using “food”, “cook”, “broiler”, “roaster”, “charbroiler” or “cooker” as the search term. If this was not successful, then a Google search using the name of the state and “air pollution regulations” “air quality regulations” and “food”, “cook”, or “cooker” would be performed. Since state permitting refers to the device being used to cook the food (“food cooker”) and not to restaurants themselves, that is why this term is used. The if there was an entry found, then the regulation was reviewed and data regarding specific regulations for that state would be recorded.

## 3. Results

### 3.1. Air Pollution Sensor Results

Characteristics of the 2016–2017 monitoring sessions are presented in Table 3. Eighteen air monitoring sessions took place in the vicinity of the restaurant of concern between June 2016 to June 2017, thirteen in the Spring (Mar–May 2017), three in the summer (June 2016 and June 2017), and two in the Winter (February 2017). The mean length of sampling sessions was 10.3 min (range 5–41 min), the mean ± SD PM_2.5_ per session was 19.8 ± 38.1 μg/m^3^ with measurements ranging from 0.48 to 339.4 μg/m^3^.

**Figure 1.**
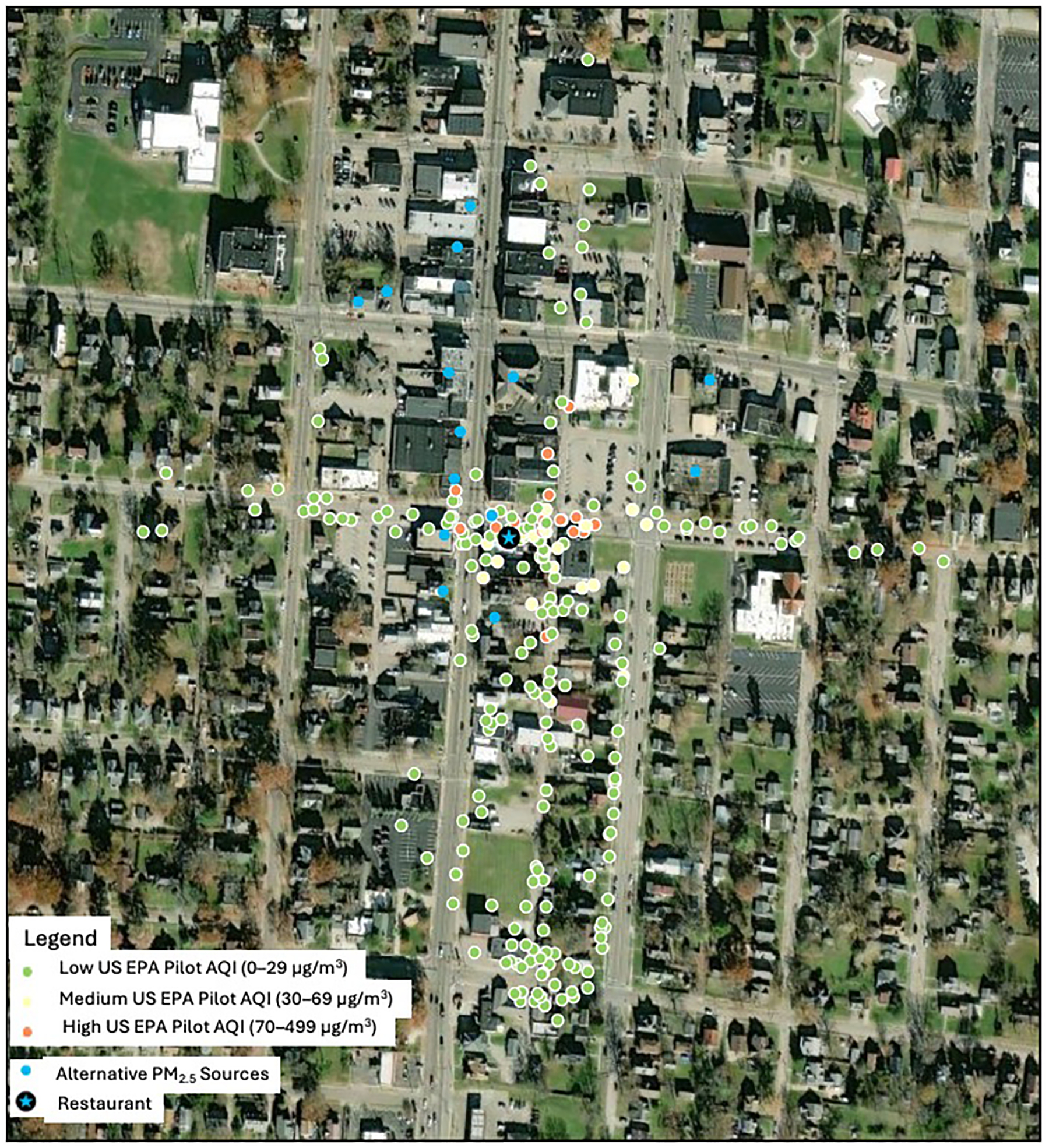
PM2.5 sensor readings by US EPA AQI Pilot 1-Minute Average (2016–2017).

Overall, 78% (14/18) of sessions had peak PM_2.5_ levels at or above 35.5 μg/m^3^, the US EPA breakpoint for “Unhealthy for Sensitive Groups,” 17% of sessions had an average PM_2.5_ level at or above 35.5 μg/m^3^. Air monitoring sessions took place as a neighborhood walkaround; there were noticeable differences in PM_2.5_ levels at different points along the walking route. Increased PM_2.5_ levels were observed closer to the restaurant (Figure 1). Of the measurements above 35.5 μg/m^3^, 63.4% of those 1 s peak measurements were recorded within 50 m of the restaurant, additionally 78.2% of the measurements above 250.5 μg/m^3^ (EPA “Hazardous”) were measured within 50 m of the restaurant of concern. When examining the 1 min PM_2.5_ measurements and comparing them with the US EPA AQI 1-Minute Pilot range, all the measurements in the “High” category (>70 μg/m^3^) were within 150 m of the restaurant (Figure 1).

**Figure 2.**
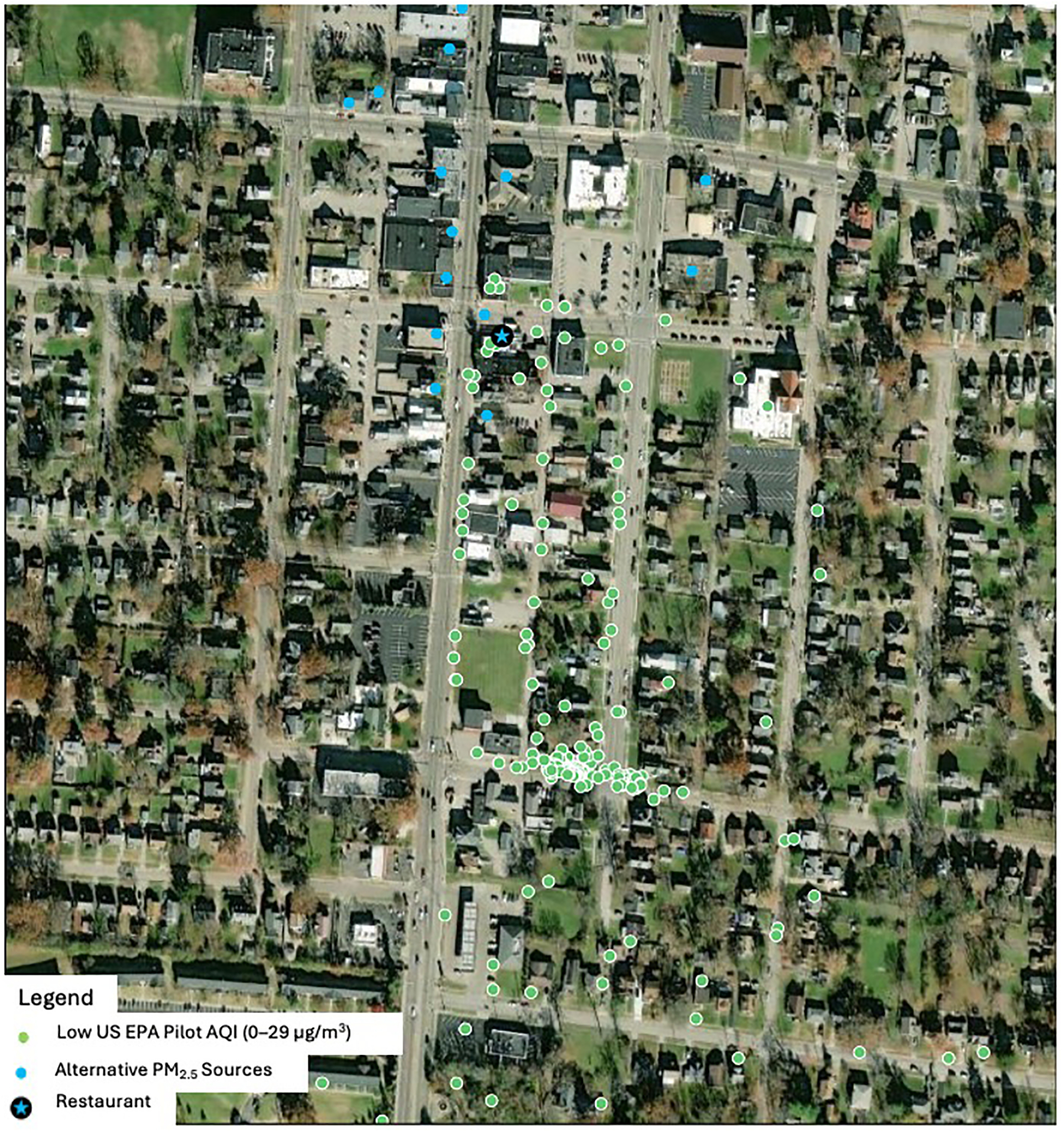
Sensor readings by US EPA AQI Pilot 1-Minute Average (2024).

Characteristics of the 2024 monitoring sessions are presented in Table 4 and Figure 2. There were 21 sessions, and these sessions overall had much lower levels of measured PM_2.5_ than in 2016–2017. Seven sessions were stationary, with the sensor mounted at roughly head height on the community member’s (DC) porch. One session was carried out with a duplicate sensor.

For the 2016–2017 sessions, distance to the restaurant and 1 min average PM_2.5_ were significantly correlated, r = −0.364, df = 236, *p* < 0.0001. With decreasing distance to the restaurant being associated with increased PM_2.5_ readings. In the 2024 sessions, distance to the restaurant was not significantly correlated with PM_2.5_ readings, r = 0.032, df = 424, *p* = 0.507. Multiple linear regression analysis was used to test the relationship between PM_2.5_ readings and distance to the restaurant, windspeed, and whether the measurement was taken upwind or downwind of the restaurant. For the 2016–2017 sessions, distance to the restaurant when adjusted for windspeed and location downwind of the restaurant was significantly negatively associated with PM_2.5_ measurements (R^2^ = 0.13, F(3, 234) = 11.90, *p* < 0.001). This relationship was explained by distance to the restaurant (β = −136.02, *p* < 0.001) whereas windspeed (β = 6.695 × 10^−4^, *p* = 0.999) and being downwind of the restaurant (β = −1.261, *p* = 0.791) were not significantly associated with PM_2.5_ measurements. For the 2024 mobile sessions, increased distance from the restaurant was associated with increased PM_2.5_ (R^2^ = 0.096, F(3, 422) = 14.967, *p* < 0.001), with distance to the restaurant (β = 1.757, *p* < 0.029), and windspeed (β = 0.339, *p* < 0.001) explaining this relationship. Being downwind of the restaurant trended towards lower PM_2.5_ measurements (β = −1.375, *p* < 0.275).

### 3.2. Government Response

The Ohio Administrative Code Chapter 3745-15-07, states that air pollution nuisances are prohibited [34]. Although they agreed that excessive smoke emissions were potentially harmful, government entities could not investigate the emissions and therefore did not enforce any restrictions, because restaurant emissions were not within their jurisdiction. However, the proprietor of the restaurant was informed of the complaints. The AirBeam not being a Federal Reference Method, nor a Federal Equivalent Method was another concern to the regulatory authorities. Ohio EPA succinctly summarized this: “Ohio EPA does not have any requirements to require controls on restaurant cookers, nor do we plan to adopt regulations to control local BBQs…” (letter from Ohio EPA, 11/8/2016). Although the restaurant may have been eligible for an exhaust scrubber through the US EPA “Making a Visible Difference in Communities” program, this program ended in early 2017.

### 3.3. Findings from Policy Review

A review of air pollution regulations across the United States indicated that only six states regulate food cookers (Alaska, California, Colorado, Massachusetts, Rhode Island, Utah) and six states (Alabama, Georgia, Hawaii, Idaho, Illinois, Wisconsin) specifically exempted cookers from air pollution regulations. Within those six states, Wisconsin and Alabama more specifically exempted outdoor cooking and non-commercial open fire. Current environmental health regulations may not protect residents living near sources of restaurant-based sources of PM_2.5_.

### 3.4. Engagement with the Restaurant Owner

During this project (2016–2025), there have been periodic interactions with the restaurant owner, the community member, and the academic partner. In these conversations, the community member was able to share concerns about smoke emissions and the restaurant owner expressed concerns about the cost of installing an emissions control system. To be a good neighbor, the restaurant owner changed the fuel for their outdoor cooker from charcoal to wood but did not recall the date that this change was made, although it was reported to be likely in 2022 or 2023.

## 4. Discussion

This project provides insights about the promise and pitfalls of advances in air pollution monitoring technology and its application to citizen science. We adapted strategies used in other communities, using a mobile, low-cost sensor to measure air pollution, but focused on a non-industrial source [35]. We consistently detected high levels of PM_2.5_ in the immediate vicinity of the restaurant, coinciding with the operation of the cooker. Barriers to mitigating the emissions include the cost of emission controls combined with no legal requirement for air permits to be issued to restaurant cookers. Positive factors included ongoing connection between the academic partner, community member, and the restaurant owner.

There were several limitations to our project. The AirBeam sensor is not as accurate as a Federal Reference Method or a Federal Equivalent Method device; however, the sensor recorded high PM_2.5_ levels consistently in the vicinity of the source. Air pollution monitoring was performed in a single neighborhood, based on the community member’s availability and may not reflect PM_2.5_ levels more generally. However, air monitoring was performed over approximately 12 months and results were consistent throughout that time. Air pollution monitoring sessions were carried out according to the community member’s availability, with most sessions taking place in the Spring season, so we could not adjust for seasonality in the measurements. There was no documentation of observations of alternative sources of PM_2.5_ collected during the sessions; thus, we lack records of background PM_2.5_ sources for the study area. However, we included in Figures 1 and 2 the location of other potential sources of PM_2.5_ in the neighborhood. Also, since the AirBeam is not weather-proof, it could not be used in rainy weather, so this is an additional limitation. Most air sampling was conducted when the restaurant cooker was active. A session conducted when the restaurant was closed demonstrated lower PM_2.5_ concentrations (Session 4 in February 2017). This suggests PM_2.5_ concentrations measured during other sessions were likely from the restaurant. We did not collect particulate matter on filters and therefore we cannot create a source apportionment model, but based on our analysis, high peak levels were consistently detected in the vicinity of the restaurant of concern. Although there were also temporal gaps in the readings available from the nearest US EPA sensor, we calculated the background PM_2.5_ by averaging the daily readings of the four closest US EPA sensors. For the 2024 sessions, we also included a stationary site in the neighborhood approximately 300 m from the restaurant.

During the 2016–2017 monitoring sessions, we found that distance from the restaurant was negatively associated with PM_2.5_ measurements, but these were not influenced by windspeed or being downwind of the restaurant. There are several possible explanations for this. We did not have real-time windspeed and direction measurements available. NOAA recorded average daily wind direction and daily sustained 5 min windspeeds from a monitoring station 18.5 km southeast of the restaurant. Therefore, we do not know the exact conditions at the location of the air pollution measurements. A similar study of short range restaurant emissions did not adjust for windspeed, wind direction, or weather conditions [11]. Future work could examine the impact of these covariates on close range restaurant emissions.

We observed that following a change in cooking fuel at the restaurant from charcoal to wood, there was a decrease in the measured PM_2.5_ levels and smoke visible near the restaurant. Typically, wood burning would produce more PM_2.5_ emissions than charcoal, in a laboratory setting [36]. It is uncertain why we have observed the opposite effect. Charcoal characteristics can influence emissions with lump charcoal (derived from hardwood) producing fewer PM_2.5_ precursor chemicals than charcoal briquettes [37]. Additionally, emissions from wood burning tend to be higher when the stove is cold, but can decrease as the when combustion conditions are optimized (warm stove, optimal air/fuel ratio) [38]. We did not collect information from the operators of the cooker to assess how the wood was being burned when measurements were taken, this is a limitation.

Public concern about the health impacts of air pollution following highly publicized and fatal incidents through the first half of the 20th Century, such as the extreme smog conditions in Donora, Pennsylvania and Meuse Valley, Belgium [39] culminated in the passage of the 1970 Clean Air Act and amendments (1977 and 1990). This reduced emissions from industrial sources and motor vehicles with a resulting trend towards improved air quality since that time. Recent and ongoing citizen science studies have helped to bring improvements in local air pollution levels as a result of industrial sources [35]. One notably successful project combined multiple community groups, academic partners, and a successful lawsuit to make improvements in air quality in a neighborhood impacted by industrial emissions [40]. Some jurisdictions do regulate food cookers, but these regulations are predominantly directed at chain-operated charbroilers so that they can comply with Clean Air Act requirements, not to address neighborhoods with potentially disproportionately high levels of exposure. Local laws do not yet reflect the more recent science regarding sources of air pollution in urban environments and therefore there were no regulatory tools with which to address the emissions identified in our project. With restaurant emissions accounting for up to 17% of particle emissions in urban areas overall and >70% of PM_2.5_ in restaurant districts, these sources of PM_2.5_ require additional attention [7,8,10,11]. Previous research indicates that grilling meat and searing meat produce the highest levels of PM_2.5_ as compared with other methods [41,42].

Although our efforts did not result in a health-protective response from local governmental bodies, the informal engagement with the restaurant owner regarding the cooking emissions did. Suggesting that under certain circumstances, this approach has potential. We identified important lessons that can inform this community-based work in the future.

1. Data from low-cost air pollution sensors may not be in a form that is readily useable. For example, raw data is averaged over 1 sec, but other intervals may be more appropriate in terms of interpreting the results for health impact.
2. Lower-cost air pollution sensors provide Citizen Scientists an assessment of air pollution exposure, but do not provide readings accurate or precise enough for regulatory action [43].
3. The sensor data is available in a “raw form”, but not all community groups who have access to a sensor will be able to process, analyze and draw conclusions from this data without help from a trained expert.
4. The “burden of proof” for our community-based project to provoke a health-protective response from local government was quite high. One would expect that it would be easier to dismiss subjective reports of citizens observing excessive smoke and odors, but less so to dismiss objective air pollution monitoring, particularly when using a sensor appropriate for the task. It seems unlikely that a community member would have access to a Federal Reference Method or a Federal Equivalent Method, even when working with an academic partner.
5. Local air pollution regulations tend to address industrial point sources and traffic-related air pollution, but rarely non-industrial sources. This may reflect environmental health risk perception. A common exposure such as cooking smoke may be seen as less risky than industrial pollution [44].
6. Due to expense, small businesses do not have access to emission mitigation resources.
7. We lack health-based short-term air pollution exposure standards. With their Village Green Project, US EPA has created pilot guidance for PM_2.5_ readings averaged over 1 min, but thus far these are not to be used for regulatory purposes [33].
8. The air pollution regulatory framework is difficult to navigate. Citizens may be concerned about air pollution, but it is unclear who has jurisdiction (state EPA, local health department, regional air quality agency, local government). All were contacted in our case; a less motivated private citizen may not have been this persistent.
9. It is cumbersome to perform analyses like ours (measuring emissions based on distance from a source) using low-cost sensors and their accompanying software which is predominantly designed for sharing results through a cloud-based system.
10. Addressing local environmental emissions can take many years of advocacy and engagement across the community-academic continuum; it can be difficult to maintain this over long periods.

Finally, with the advent of lower-cost air pollution sensors, an engaged citizenry may identify new “hyperlocal” sources of air pollution suggesting new opportunities for the improvement of public health. Environmental public health laws need updating to be able to better address these sources of air pollution. Including in regulations provisions for using low-cost sensors as “screening” devices that if they report high readings, would prompt further investigation using “gold standard” devices is one possible approach. Additionally, the utility of low-cost sensors would be improved by including user-friendly ways to obtain averages over time intervals, peak duration, or to calculate distance from a point source. Current work being carried out through US EPA to validate these lower-cost sensors against “gold standard” devices, may open the door for more effective response to excessive PM_2.5_ production from such sources in the future. Providing access to a source of funding to help businesses address these emissions, as with assistance for lead paint abatement, may help businesses and community members be more open to interventions to reduce such emissions. Improving and validating air pollution sensors is only part of the solution.

Future research should examine methods for measuring emissions from small businesses, such as restaurants and communicating these findings in a constructive way. Making just public policy in this area will require a transdisciplinary approach that includes healthcare providers, public health practitioners, policy makers, and legal professionals as these businesses face barriers to addressing air pollution emissions [45].

## 5. Conclusions

A low-cost air pollution sensor demonstrated high PM_2.5_ emissions from a local restaurant. Regulatory and public health agencies were not able to address the emissions because of a lack of jurisdiction, but informal engagement with the restaurant owner prompted action. Few states consider food cookers in their air pollution regulation. Additional work is needed to both define the scope of this problem and to create public policy to address it. A long-term community–academic partnership contributed to voluntary reductions in the source of emissions.

## Figures and Tables

**Table 1.** Program partners and partners’ objectives for the project.

Partner	Objective
Both Community Engagement Core and Community Member	Increased community engagement regarding local air pollution levels Reduce community exposure to PM_2.5_
Community Member	Educate and empower neighbors around source of air pollution Encourage government authorities to decrease emissions from the source Protect the health of those living near the source Help the restaurant reduce their emissions
Community Engagement Core	Contribute environmental health technical assistance to local communities Assist with implementing a scientifically valid air pollution monitoring plan tailored to the available resources Leverage institutional resources to help communities to achieve their environmental health goals Dissemination of findings

**Table 2.** Businesses located within a 200 m radius of the restaurant.

Description	Count
Auto Repair	1
Bank	2
Bar/Night Club	2
Barber/Salon	6
Bus stop	3
Charity	3
Church	3
Clothing	1
Construction/Maintenance	5
Funeral Home	1
Herb shop	1
Insurance	2
Library	1
Medical Office	1
Pet store	1
Post Office	1
Restaurant	7
Retail	11
School/Daycare	1
Studio (Dance, Martial Arts)	3

**Table 3.** Summary of 2016–2017 air pollution monitoring sessions.

Session	Date	Start Time	Day of Week	Length (min)	Session Mean ± SD PM_2.5_ (μg/m^3^)	Session Peak (1 min) PM_2.5_ (μg/m^3^)	Average US EPA Daily PM_2.5_ (μg/m^3^)
1	16 June 2016	8:54	Thursday	41	25.2 ± 43.3	184.8	5.60
2	17 June 2016	12:17	Friday	10	20.0 ± 33.1	66.7	8.43
3	25 February 2017	17:21	Saturday	10	18.8 ± 31.1	68.5	5.67
4	26 February 2017 *	17:27	Sunday	7	1.7 ± 0.6	2.2	6.17
5	2 March 2017	18:28	Thursday	9	5.1 ± 7.0	16.9	4.28
6	3 March 2017	8:46	Friday	6	22.0 ± 21.1	47.3	5.77
7	4 March 2017	17:50	Saturday	7	10.2 ± 14.8	26.7	8.13
8	5 March 2017	12:34	Sunday	5	55.0 ± 74.6	146.3	8.50
9	9 March 2017	18:05	Thursday	10	6.2 ± 6.6	18.0	7.80
10	11 March 2017	17:48	Saturday	12	10.3 ± 9.8	24.8	4.95
11	14 March 2017	18:45	Tuesday	8	2.4 ± 0.6	3.0	8.21
12	16 March 2017	17:18	Thursday	6	23.6 ± 29.9	81.3	11.20
13	18 March 2017	8:02	Saturday	12	52.6 ± 70.3	165.5	8.77
14	23 March 2017	17:45	Thursday	9	17.0 ± 34.0	98.6	6.42
15	30 March 2017	17:40	Thursday	11	41.2 ± 56.8	147.6	6.83
16	1 April 2017	10:39	Saturday	5	14.1 ± 13.0	43.2	4.60
17	10 April 2017	19:42	Monday	5	2.9 ± 0.6	3.2	7.78
18	8 June 2017	7:10	Thursday	10	5.3 ± 4.2	13.2	6.83

* Restaurant was closed on this day.

**Table 4.** Summary of 2024 air pollution monitoring sessions.

Session	Date	Start Time	Day of Week	Length (min)	Session Mean ± SD PM_2.5_ (μg/m^3^)	Session Peak (1 min) PM_2.5_ (μg/m^3^)	Average US EPA daily PM_2.5_ (μg/m^3^)
1	13 April 2024	14:30	Saturday	5	0.2 ± 0.4	1.0	4.6
2	14 April 2024	19:28	Sunday	29	2.0 ± 5.6	24.1	7.65
3	15 Aprill 2024	17:38	Monday	35	2.2 ± 1.3	3.2	9.85
4	16 April 2024	17:41	Tuesday	44	2.7 ± 1.3	6.0	9.13
5 *	17 April 2024	17:39	Wednesday	46	1.3 ± 1.3	5.4	4.28
6 *	18 April 2024	17:38	Thursday	59	0.0 ± 0.2	0.5	4.01
7	19 April 2024	11:57	Friday	15	0.1 ± 0.3	0.4	4.02
8	20 April 2024	15:53	Saturday	56	0.0 ± 0.1	0.2	3.4
9	21 April 2024	7:42	Sunday	43	0.2 ± 0.4	0.8	6.0
10	22 April 2024	17:37	Monday	38	0.7 ± 0.7	1.6	8.8
11#	23 April 2024	12:28	Tuesday	10	1.5 ± 0.7	2.2	7.7
12 *	23 April 2024	17:36	Tuesday	10	0.9 ± 0.8	2.8	7.7
13	24 April 2024	18:49	Wednesday	84	0.4 ± 1.3	8.9	5.55
14 *	26 April 2024	17:34	Friday	35	1.5 ± 0.8	2.8	7.7
15 *	27 April 2024	7:05	Saturday	31	10.3 ± 2.9	13.5	12.68
16	27 April 2024	16:13	Saturday	45	7.7 ± 1.0	8.9	12.68
17 *	28 April 2024	16:04	Sunday	45	3.3 ± 1.2	5.4	8.85
18 *	29 April 2024	17:35	Monday	45	3.2 ± 1.2	4.3	9.13
19	30 April 2024	17:45	Tuesday	100	0.0 ± 0.1	0.2	4.06
20	2 May 2024	11:12	Thursday	14	7.3 ± 1.1	9.2	9.3
21	3 May 2024	10:40	Friday	14	6.4 ± 0.9	7.1	9.98

* Stationary session.

# Mobile session with duplicate sensor.

## Data Availability

The data presented in this study are available on request from the corresponding author due to it containing high resolution location data.

## Abbreviations

The following abbreviations are used in this manuscript:

US EPA: United Stated Environmental Protection Agency
PM_2.5_: Particulate matter < 2.5 μm in diameter
AQI: Air Quality Index
EPA: Environmental Protection Agency
NIEHS: National Institute for Environmental Health Sciences
CEC: Community Engagement Core
